# Successful Medical Management of an Acute Ascending Aortic Dissection After Coronary Artery Bypass Graft Surgery

**DOI:** 10.7759/cureus.17086

**Published:** 2021-08-11

**Authors:** Marc T Zughaib, Harshil Patel, Marcel E Zughaib

**Affiliations:** 1 Internal Medicine, Ascension Providence Hospital/Michigan State University College of Human Medicine (MSUCHM), Southfield, USA; 2 Cardiology, Ascension Providence Hospital, Southfield, USA; 3 Cardiology, Ascension Providence Hospital/Michigan State University College of Human Medicine (MSUCHM), Southfield, USA

**Keywords:** coronary artery bypass grafting(cabg), type a aortic dissection, adult cardiology, cardiothoracic surgery, type a acute aortic dissection

## Abstract

Aortic dissection is an acute and life-threatening disease entity. Mortality rates increase every hour after the presentation. Typical treatment includes medical management of blood pressure and heart rate control followed by prompt transfer to an operating room for surgical repair. We present a case of medically managed Stanford type A aortic dissection in a postoperative coronary artery bypass graft (CABG) patient.

A 77-year-old man with a past medical history of hypertension and hyperlipidemia presented after an outpatient nuclear stress test demonstrated a reversible inferior wall defect. He was subsequently referred to a cardio-thoracic surgeon and underwent coronary artery bypass graft (CABG) surgery. Three weeks later, the patient presented to the emergency department complaining of a productive cough, nausea, vomiting, and fever. He was diagnosed with sepsis secondary to pneumonia. A CT chest demonstrated a new 3.9 cm long segment of dissection in the ascending thoracic aorta. Due to postoperative recovery from recent CABG, a decision was made to treat the ascending thoracic aortic dissection (Stanford type A) medically. He was advised to continue intensive antihypertensive medications and close follow-up with a cardiologist and cardiothoracic surgeon on an outpatient basis. Subsequent follow-up CT chest angiography at one month, four months, and 12 months later did not demonstrate the progression of the ascending aortic dissection.

Decisions to deviate from the usual care should best be taken in a multidisciplinary team approach. Patients should clearly be informed about the rationale behind these complex decisions.

## Introduction

Aortic dissection is an uncommon phenomenon, but typically presents acutely and could be life-threatening [1]. Aortic dissections are classified according to the DeBakey and Stanford classifications. The Stanford classification, which is used more commonly, describes the dissections involving ascending aorta as type A and all other dissections in the aorta as type B [2]. Type A dissection is more common than type B [3]. Acute Stanford type A aortic dissections are surgical emergencies. Mortality rates are noted to be 1-2% per hour after symptom onset without surgical intervention [4]. Typical treatment includes medical management of blood pressure and heart rate control followed by prompt transfer to an operating room for surgical repair. After surgical repair, patients are maintained on lifelong therapy to reduce blood pressure with a target pressure less than 120/80 mmHg [5]. In contrast, type B dissections are generally managed medically, with surgical intervention reserved for patients who develop hemodynamic instability or complications related to the dissection [6].

Relatively common risk factors for developing an aortic dissection include uncontrolled hypertension, connective tissue disorders, bicuspid aortic valve, and trauma. Aortic dissections following cardiac catheterization as well as coronary artery bypass grafting (CABG) are rare. Tsai et al. found the incidence of ascending aortic dissection to be one out of 2,723 patients treated with CABG from a single institution [7]. We report a case of a recent CABG patient presenting with a Stanford type A aortic dissection successfully managed medically rather than surgically.

## Case presentation

A 77-year-old man with hypertension and hyperlipidemia underwent an outpatient nuclear stress test which demonstrated a reversible inferior wall defect. He subsequently underwent cardiac catheterization diagnosing severe multivessel atherosclerotic disease. A referral was placed to a cardio-thoracic surgeon for coronary artery bypass graft (CABG) surgery. A chest CT scan obtained during pre-CABG evaluation demonstrated a mild ascending aortic root dilation measuring 4.1 x 4 cm, without any evidence of dissection. The patient subsequently underwent CABG without any intra-procedural or immediate post-procedural complications. He was discharged home in a hemodynamically stable condition on the fifth postoperative day.

**Figure 1:**
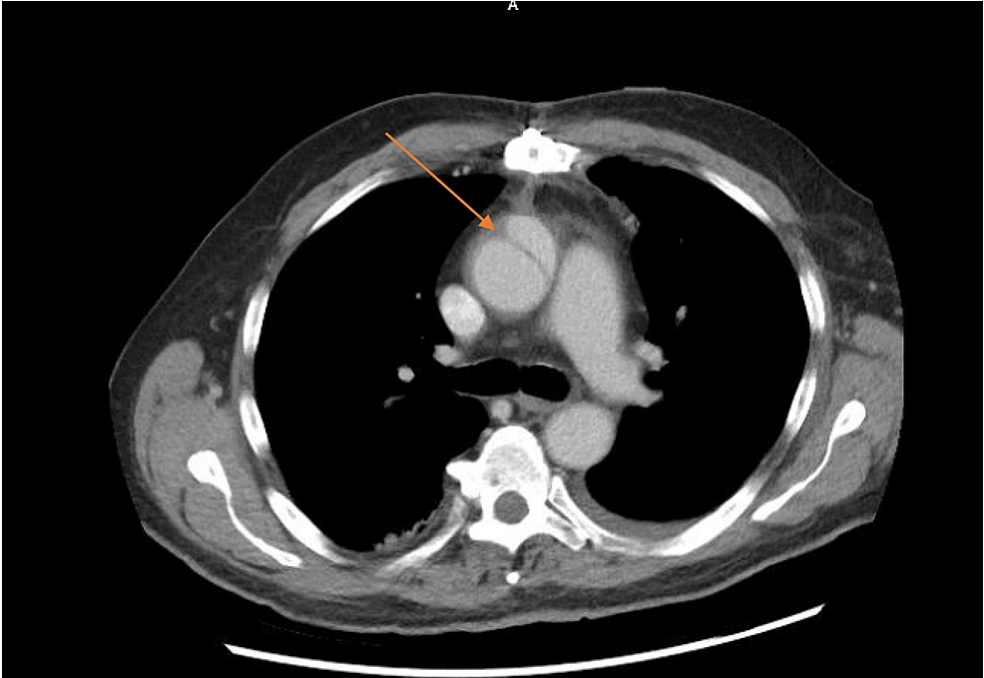
CT chest demonstrating a new dissection of the ascending thoracic aorta with false lumen adjacent to the true lumen. Arrow in figure pointing to dissection.

**Figure 2:**
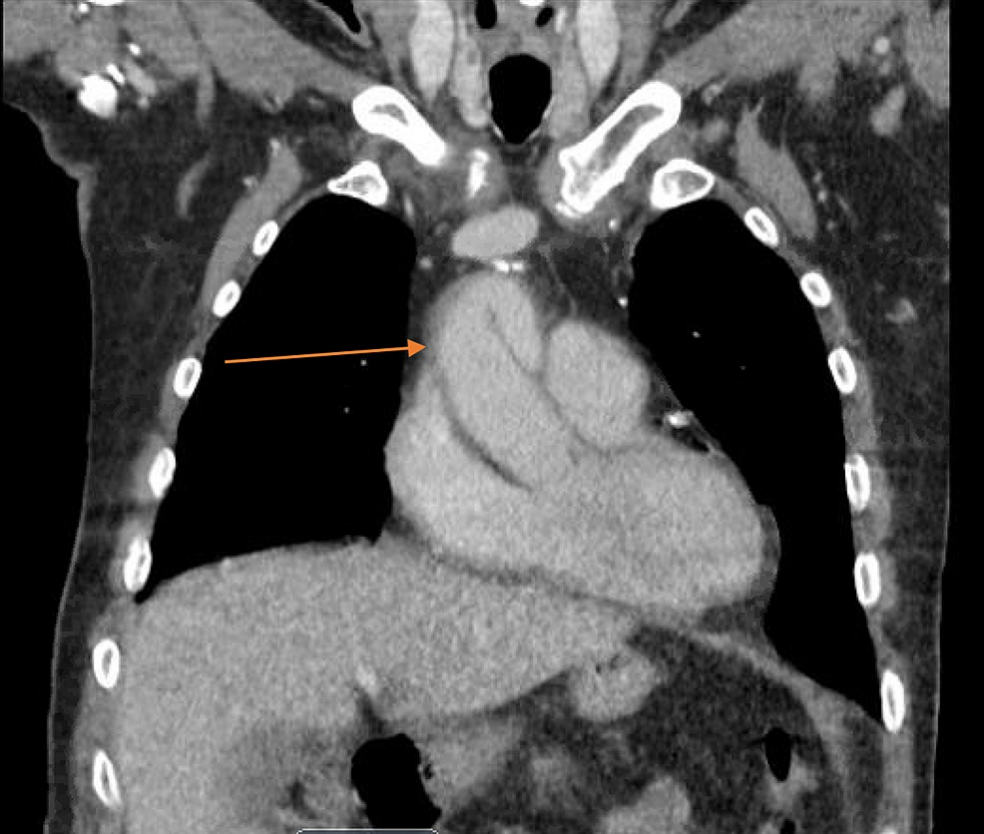
CT chest demonstrated a new 3.9 cm long segment of dissection of the ascending thoracic aorta, originating 3 cm distal to the aortic root. Arrow in figure pointing to dissection.

Three weeks later, the patient presented to the emergency department complaining of a productive cough, nausea, vomiting, and fever. Based on physical examination, laboratory and imaging studies, he was diagnosed with sepsis secondary to pneumonia. A CT chest performed during initial evaluation for pneumonia in the ER redemonstrated the ascending thoracic aortic root dilation, as well as a new 3.9 cm long segment of dissection of the ascending thoracic aorta, originating 3 cm distal to the aortic root (Figures 1, 2). The dissection did not involve recently grafted vessels. The new dissection was felt to be iatrogenic secondary to the recent CABG surgery. Treatment options along with their benefits and risks were discussed in detail with the patient. Medical management of the new dissection was agreed upon between the patient and the team of multidisciplinary physicians. This was a deviation from standard practice, albeit the most pragmatic approach, and this was communicated to the patient clearly.

The patient was started on metoprolol and losartan, with a blood pressure goal of less than 130 mmHg systolic. The patient remained hemodynamically stable throughout this hospitalization. A repeat CT scan was performed nine days after presentation, which demonstrated an unchanged type A dissection. The patient’s sepsis secondary to pneumonia was managed successfully with IV ceftriaxone and vancomycin. On day 12 of hospitalization, the patient experienced hypoxia and difficulty breathing. He was diagnosed with a thrombosis in the left greater saphenous vein, as well as subsegmental pulmonary embolism. It was determined he was not a candidate for catheter-directed pharmaco-mechanical thrombolysis by the pulmonary/critical care team. He was managed with heparin infusion thereafter. On day 17, he was discharged with Lovenox subcutaneous, and an order for a follow-up CT scan chest in one month to evaluate for any worsening of the aortic dissection. He was advised to continue losartan 25 mg once a day and metoprolol 100 mg BID with close follow-up with a cardiologist and cardiothoracic surgeon on an outpatient basis. Subsequent follow-up CT chest angiography at one month, four months, and 12 months did not demonstrate the progression of the ascending aortic dissection. 

## Discussion

Aortic dissections are relatively rare phenomena, with a reported incidence of 5-10/100,000 person-years [5]. Additionally, the incidence of an acute aortic dissection after thoracic surgery has been reported to be 0.1-0.2% [6,8,9]. A previous study by Eitz et al. demonstrated that of the 22,000 patients who underwent CABG, 12 patients subsequently presented with an aortic dissection [10]. Only five of these patients presented within the first year post-CABG, with an average time to dissection of 2.5 years.

 A multivariate analysis by Galloway et al. showed that older age, ascending aortic aneurysm extending into the descending aorta as well as the involvement of the aortic arch are predictors of higher operative mortality [11]. Our patient presented with aortic dissection in the previously dilated segment which makes surgical management the usual treatment [1,2]. Patients having undergone a recent major surgical procedure like coronary artery bypass graft are certainly considered at high risk if another thoracotomy is being contemplated. Patients presenting with acute Stanford type A aortic dissections that extend proximally to involve the aortic root, aortic valve, or the coronary arteries would probably be better managed by surgical treatment. However, there have been some case reports of proximal extension of aortic dissection into the coronary tree responsible for ischemia and poor outcomes [12,13]. Stanford type A dissections that are relatively more distal in the ascending aorta, in a hemodynamically stable patient may be considered to be managed by aggressive medical therapy with titration to maximal dosing of beta-blockers was done in our patient. This is especially true if the size of the dissection is not too large and if the risks associated with surgical treatment in a patient with recent CABG are significant. While there are general guidelines for managing acute ascending aortic dissections, there are no specific recommendations regarding treatment options for Stanford type A dissections in the immediate postoperative period after a major intrathoracic surgery like coronary artery bypass graft [2]. Our case redemonstrates that practicing medicine is an art where one size may not fit all, and individualized therapies lead to favorable patient outcomes. 

## Conclusions

Type A aortic dissections are considered surgical emergencies. In patients with recent major intra-thoracic/intra-abdominal surgical procedures, the risk of open thoracotomy and surgical treatment of dissections in terms of adverse outcomes may be unacceptably high. Medical management with aggressive blood pressure control and close surveillance may be a safer and effective alternative for these patients, particularly in the setting of a small dissection not involving the aortic valve or coronary arteries. Such decisions should best be taken in a multidisciplinary team approach. Patients should clearly be informed about the rationale for the deviation of treatment from the standard of care. Finally, more studies are needed to establish specific guidelines to manage Stanford type A aortic dissections in patients who are considered a high surgical risk.
